# Supplementary material for: Instability in Computational Models of Vascular Smooth Muscle Cell Contraction
Source: Ann Biomed Eng. 2024 Jun 29;52(9):2403–16. doi: 10.1007/s10439-024-03532-x (PMC11329416; doi:10.1007/s10439-024-03532-x)
Supplement: Supplementary file 1 — Supplementary file1 (PDF 307 kb) [file 10439_2024_3532_MOESM1_ESM.pdf]

# Instability in computational models of vascular smooth muscle cell contraction

Alessandro Giudici*, Jason M Szafron*, Abhay B Ramachandra, Bart Spronck

*Authors contributed equally.

# Supplementary Information

**Corresponding author:**

Bart Spronck, PhD

Department of Biomedical Engineering
Cardiovascular Research Institute Maastricht (CARIM)
Maastricht University
Universiteitssingel 40, Room C5.578A
Maastricht, 6229 ER
The Netherlands
b.spronck@maastrichtuniversity.nl

## S1. Theoretical analysis in a thin-walled cylinder

For a thin-walled cylindrical tube (i.e., $b - a \ll a$), Eq. 2 can be approximated to

$$P \cong (\bar{t}_{\theta\theta} - \bar{t}_{rr}) \frac{h}{r_\mathrm{m}} \,, \tag{S1}$$

where $h = b - a$ and $r_\mathrm{m} = (a + b)/2$ are the wall thickness and the mid-wall radius, and $\bar{\boldsymbol{t}}$ is the average Cauchy wall stress tensor across the wall thickness. Note that, under the assumption that $\bar{t}_{rr} = P/2$, Eq. S1 is equivalent to the well-known Laplace equation

$$P \cong \bar{t}_{\theta\theta} \frac{h}{a} \,. \tag{S2}$$

As done for the thick-wall derivation, the Cauchy stress in Eq. S2 can be split into an active and a passive contribution, to yield

$$P \cong \left(\bar{t}_{\theta\theta}^{\mathrm{pas}^*} - \bar{t}_{rr}^{\mathrm{pas}^*}\right) \frac{h}{r_\mathrm{m}} + \bar{t}_{\theta\theta}^{\mathrm{act}^*} \frac{h}{r_\mathrm{m}} \,, \qquad \text{and} \tag{S3}$$

$$\Gamma^\mathrm{act} \cong \bar{t}_{\theta\theta}^{\mathrm{act}^*} \frac{h}{r_\mathrm{m}} \,. \tag{S4}$$

### S1.1. Constant stress active contraction models

We then proceed with analysing the thin-wall equivalent of the three constant active stress models described above for the thick-wall case. For a constant active stress in the current configuration, $\bar{t}_{\theta\theta}^{\mathrm{act}^*} = T_\mathrm{c}$, so that

$$\Gamma^\mathrm{act} \cong T_\mathrm{c} \frac{h}{r_\mathrm{m}} \,. \tag{S5}$$

Knowing that, if $b - a \ll b + a$, $\ln(b/a) \cong 2(b - a)/(b + a)$, it becomes apparent that Eq. 7 can be recovered from Eq. S5 and that the constant Cauchy active stress model preserves the features discussed for the thick-walled case.

Similarly, for a constant active stress in the intermediate configuration, $\bar{t}_{\theta\theta}^{\mathrm{act}^*} = T_\mathrm{i} \bar{\lambda}_\theta$ and

$$\Gamma^\mathrm{act} \cong T_\mathrm{i} \frac{h}{R_\mathrm{m}} = T_\mathrm{i} \frac{H}{\lambda_z r_\mathrm{m}} \,, \tag{S6}$$

with $H = B - A$ and $h = \frac{H R_\mathrm{m}}{\lambda_z r_\mathrm{m}}$ due to incompressibility. Multiplying and dividing Eq. S6 by $R_\mathrm{m} + \sqrt{\lambda_z} r_\mathrm{m}$ we obtain

$$\Gamma^{\mathrm{act}} \cong \frac{T_{\mathrm{i}}}{\sqrt{\lambda_z}}\left[\frac{H\left(R_{\mathrm{m}}+\sqrt{\lambda_z}r_{\mathrm{m}}\right)}{\sqrt{\lambda_z}r_{\mathrm{m}}\left(R_{\mathrm{m}}+\sqrt{\lambda_z}r_{\mathrm{m}}\right)}\right] = \frac{T_{\mathrm{i}}}{\sqrt{\lambda_z}}\left[\frac{H+\sqrt{\lambda_z}h}{R_{\mathrm{m}}+\sqrt{\lambda_z}r_{\mathrm{m}}}\right] = \frac{2T_{\mathrm{i}}}{\sqrt{\lambda_z}}\left[\frac{\left(B+\sqrt{\lambda_z}b\right)-\left(A+\sqrt{\lambda_z}a\right)}{\left(B+\sqrt{\lambda_z}b\right)+\left(A+\sqrt{\lambda_z}a\right)}\right]. \tag{S7}$$

Once more (see Eq. S5), if, as in a thin-walled cylinder, $\left(B+\sqrt{\lambda_z}b\right)-\left(A+\sqrt{\lambda_z}a\right) \ll \left(B+\sqrt{\lambda_z}b\right)+\left(A+\sqrt{\lambda_z}a\right)$, then

$$2\left[\frac{\left(B+\sqrt{\lambda_z}b\right)-\left(A+\sqrt{\lambda_z}a\right)}{\left(B+\sqrt{\lambda_z}b\right)+\left(A+\sqrt{\lambda_z}a\right)}\right] \cong \ln\left(\frac{B+\sqrt{\lambda_z}b}{A+\sqrt{\lambda_z}a}\right), \tag{S8}$$

and Eq. S7 is equivalent to Eq. 11.

Finally, for a constant active stress in the reference configuration, $\bar{t}_{\theta\theta}^{\mathrm{act}^*} = T_{\mathrm{r}}\bar{\lambda}_{\theta}^{2}$, leading to

$$\Gamma^{\mathrm{act}} \cong T_{\mathrm{r}}\frac{r_{\mathrm{m}}h}{R_{\mathrm{m}}^{2}} = \frac{T_{\mathrm{r}}}{\lambda_z}\frac{H}{R_{\mathrm{m}}} = \frac{2T_{\mathrm{r}}}{\lambda_z}\frac{B-A}{B+A} \cong \frac{T_{\mathrm{r}}}{2\lambda_z}\ln\left(\frac{B^2}{A^2}\right), \tag{S9}$$

with $\ln\left(\frac{B^2}{A^2}\right) \cong 4\frac{B-A}{B+A}$ if $B-A \ll B+A$.

### S.1.2. Physiologically motivated models

With Eqs. S5, S6, and S9, we have shown that thin-walled modelling does not alter the behaviour of the considered constant active stress models, including the potential development of instability. As more complex models are typically equivalent to the superimposed contribution of these simpler formulations, the same argument holds also in those more complex cases. For a thin-walled vessel, the Rachev and Hayashi's active component of the intraluminal pressure is

$$\Gamma^{\mathrm{act}} = \frac{T_{\mathrm{Rv}}H}{\lambda_z r_{\mathrm{m}}}\left[1-\frac{\lambda_{\mathrm{m}}^{2}}{(\lambda_{\mathrm{m}}-\lambda_0)^2}\right] + \frac{2T_{\mathrm{Rv}}H}{R_{\mathrm{m}}\lambda_z}\frac{\lambda_{\mathrm{m}}}{(\lambda_{\mathrm{m}}-\lambda_0)^2} - \frac{T_{\mathrm{Rv}}hr_{\mathrm{m}}^{2}}{R_{\mathrm{m}}^{3}}\frac{1}{(\lambda_{\mathrm{m}}-\lambda_0)^2}. \tag{S10}$$

Recalling Eqs. S7 and S9 and the thin-walled assumption, Eq. S10 can be rewritten as

$$\Gamma^{\mathrm{act}} = \frac{T_{\mathrm{Rv}}}{\sqrt{\lambda_z}(\lambda_{\mathrm{m}}-\lambda_0)^2}\left[(\lambda_{\mathrm{m}}-\lambda_0)^2-\lambda_{\mathrm{m}}^{2}\right]\ln\left(\frac{B+\sqrt{\lambda_z}b}{A+\sqrt{\lambda_z}a}\right) + \frac{T_{\mathrm{Rv}}}{\lambda_z(\lambda_{\mathrm{m}}-\lambda_0)^2}\left[\lambda_{\mathrm{m}}\ln\left(\frac{B^2}{A^2}\right)\right] - \frac{T_{\mathrm{Rv}}hr_{\mathrm{m}}^{2}}{R_{\mathrm{m}}^{3}}\frac{1}{(\lambda_{\mathrm{m}}-\lambda_0)^2}. \tag{S11}$$

To recover Eq. 19, we are left with the task of proving that

$$-\frac{T_{\mathrm{Rv}}}{(\lambda_{\mathrm{m}}-\lambda_0)^2}\frac{hr_{\mathrm{m}}^2}{R_{\mathrm{m}}^3}=\frac{T_{\mathrm{Rv}}}{\lambda_z(\lambda_{\mathrm{m}}-\lambda_0)^2}\left[\frac{b}{B}-\frac{a}{A}-\frac{\ln\left(\frac{B+\sqrt{\lambda_z}b}{A+\sqrt{\lambda_z}a}\right)}{\sqrt{\lambda_z}}\right]. \tag{S12}$$

Using Eqs. S6–S8, Eq. S12 can be rewritten as

$$-\frac{T_{\mathrm{Rv}}}{(\lambda_{\mathrm{m}}-\lambda_0)^2}\frac{hr_{\mathrm{m}}^2}{R_{\mathrm{m}}^3}=\frac{T_{\mathrm{Rv}}}{\lambda_z(\lambda_{\mathrm{m}}-\lambda_0)^2}\left[\frac{bA-aB}{AB}-\frac{h}{R_{\mathrm{m}}}\right]. \tag{S13}$$

Then, substituting $R_{\mathrm{m}}=\frac{B+A}{2}$ and $h=b-a$ and opportunely rearranging yield

$$-\frac{T_{\mathrm{Rv}}}{(\lambda_{\mathrm{m}}-\lambda_0)^2}\frac{hr_{\mathrm{m}}^2}{R_{\mathrm{m}}^3}=-\frac{T_{\mathrm{Rv}}}{(\lambda_{\mathrm{m}}-\lambda_0)^2}\frac{H}{\lambda_z 2R_{\mathrm{m}}}\frac{(bA+aB)}{AB}. \tag{S14}$$

Approximating $\frac{bA+aB}{AB}\cong\frac{2r_{\mathrm{m}}}{R_{\mathrm{m}}}$ which is reasonable for a thin-walled cylinder then leads to

$$-\frac{T_{\mathrm{Rv}}}{(\lambda_{\mathrm{m}}-\lambda_0)^2}\frac{hr_{\mathrm{m}}^2}{R_{\mathrm{m}}^3}=-\frac{T_{\mathrm{Rv}}}{(\lambda_{\mathrm{m}}-\lambda_0)^2}\frac{Hr_{\mathrm{m}}}{\lambda_z R_{\mathrm{m}}^2}, \tag{S15}$$

which is true given that $\frac{hr_{\mathrm{m}}}{R_{\mathrm{m}}}=\frac{H}{\lambda_z}$ because of incompressibility.

As seen in Eq. 23, the Zulliger et al. active stress model reduces to the subtraction between a constant 1st Piola-Kirchhoff and a constant Cauchy stress term. In the thin-walled form, the active pressure is

$$\Gamma^{\mathrm{act}}=\frac{S_2T_{\mathrm{Zr}}\lambda_{\mathrm{pre}}h}{R_{\mathrm{m}}}-\frac{S_2T_{\mathrm{Zr}}h}{r_{\mathrm{m}}}, \tag{S16}$$

which recalling Eqs. S6–S8 and S5, for a thin-walled cylinder approximates well

$$\Gamma^{\mathrm{act}}=\frac{S_2T_{\mathrm{Zr}}\lambda_{\mathrm{pre}}}{\sqrt{\lambda_z}}\ln\left(\frac{B+\sqrt{\lambda_z}b}{A+\sqrt{\lambda_z}a}\right)-S_2T_{\mathrm{Zr}}\ln\left(\frac{b}{a}\right), \tag{S17}$$

i.e., the thick wall expression of $\Gamma^{\mathrm{act}}$.

The thin-walled expression of the Franchini et al.'s model active pressure (with $m_1=2$) is

$$\Gamma^{\mathrm{act}}=\frac{2T_{\mathrm{Fr,1}}r_{\mathrm{m}}h}{R_{\mathrm{m}}^2}(1-\alpha_1-\beta_1)+\frac{2T_{\mathrm{Fr,1}}r_{\mathrm{m}}^3h}{R_{\mathrm{m}}^4}(\alpha_1+2\beta_1)-2T_{\mathrm{Fr,1}}\beta_1\frac{hr_{\mathrm{m}}^5}{R_{\mathrm{m}}^6}. \tag{S18}$$

Recovering Eq. 31 from Eq. S18 is not trivial, especially for the rightmost term in Eq. S18 which originates from the integral $\int_a^b\frac{\lambda_\theta^6}{r}\mathrm{d}r$. However, the graphical comparison in **Figure 3F** proves the good agreement between the thick- and thin-walled formulations.

## Supplementary tables

**Table S1** – Four-fibre family strain energy density function parameters (obtained by refitting the modelled response of mice in the C57BL/6J control group in Spronck et al. [5]).

| $\mu$ [kPa] | $k_1^1$ [kPa] | $k_2^1$ [-] | $k_1^2$ [kPa] | $k_2^2$ [-] | $k_1^{3,4}$ [kPa] | $k_2^{3,4}$ [-] | $\alpha^{3,4}$ [°] |
|---|---|---|---|---|---|---|---|
| 26.9 | 4.5 | 0.36 | 11.6 | 0.05 | 0.9 | 1.04 | $\pm$33 |

## Supplementary figures

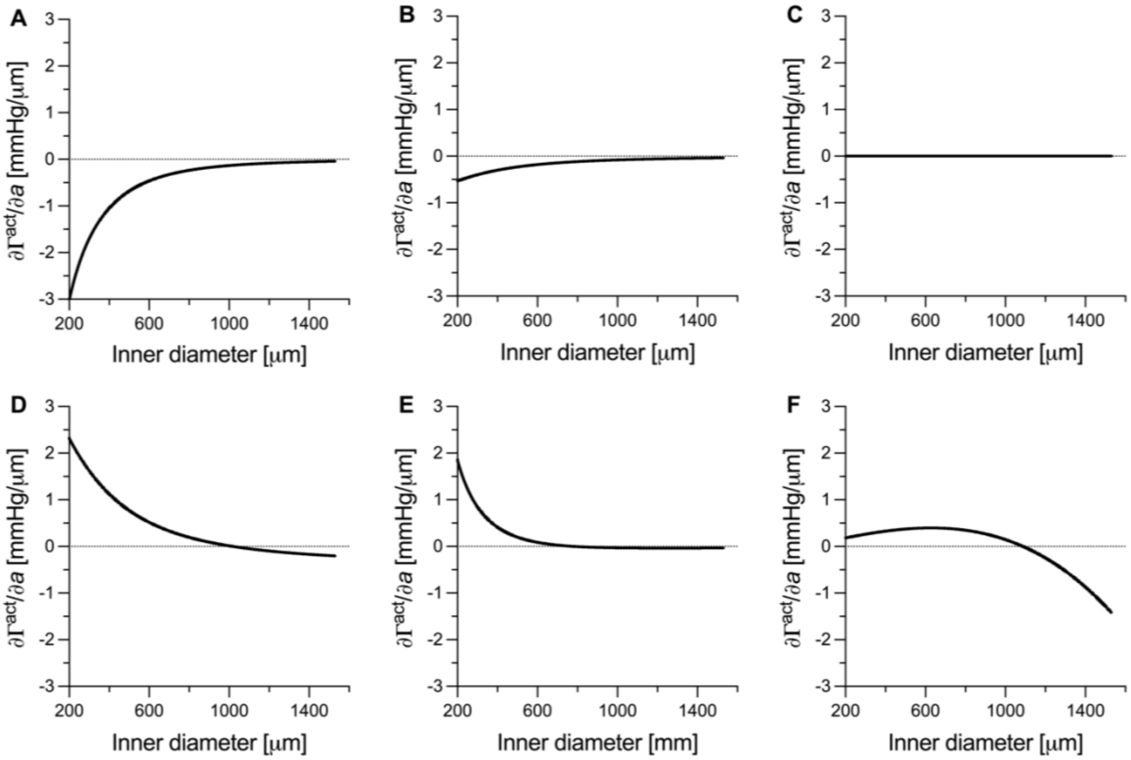


**Figure S1** – Slope of the active load bearing ($\Gamma^{\mathrm{act}}$) – inner radius ($a$) relationship of the six active stress model used to capture the contractile behaviour of the mouse thoracic aorta: constant Cauchy model (Panel A), costant 1st Piola-Kirchhoff (PK) model (Panel B), constant 2nd PK model (Panel C), Rachev model (Panel D), Zulliger model (Panel E), and Franchini model (Panel F). The modelled contractile behaviour may show instability whenever the slope $\partial\Gamma^{\mathrm{act}}/\partial a$ is negative, depending on the slope of the passive load bearing ($\Gamma^{\mathrm{pas}}$) – inner radius ($a$) relationship.
